# Trends of mortality due to oral and oropharyngeal cancers in Uruguay from 1997 to 2014

**DOI:** 10.4317/medoral.23457

**Published:** 2020-02-10

**Authors:** Maria Laura Cosetti-Olivera, Amanda R. Da Cunha, Taiane S. Prass, Marco Antonio T. Martins, Fernando N. Hugo, Manoela D. Martins

**Affiliations:** 1 Universidade Federal do Rio Grande do Sul, Programa de Pós-Graduação em Odontologia, RS, Brasil; 2 Universidad de la República, Facultad de Odontologia, Patologia y Estomatologia, Montevideo, Uruguay; 3 Universidade Federal do Rio Grande do Sul, Departamento de Estatística, RS, Brasil

## Abstract

**Background:**

To analyze the trends of oral and oropharyngeal cancer mortality in Uruguay between 1997 and 2014 according to sex and age groups and its possible association with sociodemographic factors.

**Material and Methods:**

A time-series ecological study using secondary data was performed. The data about mortality due to oral and oropharyngeal cancers were obtained from the Statistics Vitals Department of the Public Health Ministry of Uruguay. To estimate the mortality trends of the historical series, by sex, anatomical site and age groups, linear regressions generated by the Prais-Winsten procedure were used.

**Results:**

The analysis of mortality trends for oral cavity and oropharyngeal cancers in Uruguay indicated that the global mortality rate was stable over the studied period. The women's mortality rate increased from 0.51 per 100,000 in 1997 to 0.65 per 100,000 in 2014 while for men, rates per 100,000 went from 3.22 in 1997 to 2.20 per 100,000 in 2014. Mortality from oral cancer in men decreased between 1997 and 2014. Mortality by oropharyngeal cancer, irrespective of sex, remained stable. Analysis by cancer site revealed decreasing trends tumors situated in the base of the tongue and gum. Years of education, unemployment, smoking and Gini index were not associated with mortality trends.

**Conclusions:**

The overall mortality from oral and oropharyngeal cancer in Uruguay has remained constant in the period between 1997 and 2014. Oral cancer mortality decreased in men and increased in women and decreased at the base of the tongue. It’s necessary to continue monitoring the behavior of these diseases.

** Key words:**Mortality, oral cancer, oropharyngeal cancer, tongue cancer.

## Introduction

In 2018, more than 887,659 cases and 453,307 deaths from Head and neck cancers (HNC) were estimated worldwide (1). Despite advances in treatment protocols, the overall survival rates are low, most patients die within five years of diagnosed disease. Mortality associated with HNC, age-adjusted rates, varies between 3-4 per 100,000 in men and 1.5 to 2 per 100,000 in women (2). HNC represent a major public health problem in less developed countries and several variations in disease trends have been reported worldwide (3-6).

In the European Union, mortality from oral and oropharyngeal cancer decreased in both men and women between 1992 and 2002, with a greater decrease observed in men (7). In Germany, there was a significant increase of 1.4% in mortality from oral and oropharyngeal cancer in women in the last decade, while in men a slight decrease was observed (4). Mortality between 1990 and 2006 decreased by 1.83% per year in men and by 0.64% in women in the United States (8). Australia and Canada also showed a decrease in their global mortality rate from oral and oropharyngeal cancer in recent decades (5,6). Some studies showed a similar trend in South America (9-13). Perdomo *et al*. 2016 demonstrate a decrease in mortality trends by oral oropharyngeal cancers in most countries of South America (9).

Uruguay is in the southern Atlantic region of South America, with a population of 3,300,000 inhabitants approximately, of which about half live in the capital city, Montevideo (14). According to the United Nations Development Programme (UNDP), Uruguay has a very high Human Development Index, with a value of 0.804. In addition, the Uruguayan government implemented public policies regarding tobacco in 2005 which lead to a reduction in the prevalence of smoking in the general population (15). Until now, no study was developed in Uruguay analyzing mortality trends due to oral and oropharyngeal cancers. Thus, the aim of this present study was to analyze the trends of oral and oropharyngeal cancer mortality in Uruguay between 1997 and 2014 and its association with key sociodemographic factors.

## Material and Methods

This is a time-series ecological study with secondary data. The data on mortality due to oral and oropharyngeal cancers were obtained from the Statistics Vitals Department of Public Health Ministry of Uruguay and included the period between 1997 and 2014. The year 2011 was not analyzed, since no data was available due to problems in the national register. The anatomical sites of the neoplasms were determined by the International Classification of Diseases, according to the codes used in the 10th revision (ICD-10) and included: C00 – Malignant Neoplasm of Lip; C01 – Malignant Neoplasm of the Base of the Tongue; C02 – Malignant Neoplasm of Other Unspecified Parts of Tongue; C03 – Malignant Neoplasm of the Gums; C04 – Malignant Neoplasm of the Floor of the Mouth; C05 – Malignant Neoplasm of the Palate; C06 – Malignant Neoplasm of Other Unspecified Parts of the Mouth; C07 – Malignant Neoplasm of the Parotid Gland; C08 – Malignant Neoplasm of Other Unspecified Major Salivary Glands; C09 – Malignant Neoplasm of the Tonsil and C10 – Malignant Neoplasms of the Oropharynx.

Geographically, Uruguay is composed of 19 departments or provinces: Artigas, Canelones, Cerro Largo, Colonia, Durazno, Flores, Florida, Lavalleja, Maldonado, Montevideo, Paysandu, Rio Negro, Rivera, Rocha, Salto, San Jose, Soriano, Tacuarembo and Treinta y Tres. Mortality trends were analyzed for each of them.

Mortality trends were adjusted and analyzed by sex and age groups (15 to 19 years; 20 to 29 years; 30 to 39 years; 40 to 49 years; 50 to 59 years; 60 to 69 years; 70 to 79 years and 80 years or more) using the direct method (16) and referencing the world population in the year 2011. Deaths by oral and oropharyngeal cancers were analyzed jointly and also separately. Further, associations between cancers and years of education, unemployment, smoking and the Gini index, for which data were available in the period between 2006 and 2015, were tested. The proportion of unemployed people, the Gini index and the proportion of smokers were considered to be continuous variables. Multicollinearity analysis identified the presence of multicollinearity among most of the studied covariates in both men and women, indicating that they are not associated with mortality. A mixed model, also known as a random coefficient model, was used for the statistical analysis of the relationship between the mortality rates due to oral and oropharyngeal cancer with the covariates. The age group of 15 to 19 years was excluded from the analysis because it included only one death before 2006. To estimate the mortality trends in the historical series, according to sex, anatomical site and age group, linear regressions generated by the Prais-Winsten method were used (17). This procedure allowed us to classify mortality rates and annual averages as increasing (*p*<0.05 and a positive regression coefficient), decreasing (*p* <0.05 and a negative regression coefficient) or sTable (*p*>0.05). The “R”, version R-3.4.3 software, was used.

## Results

**Figure 1:**
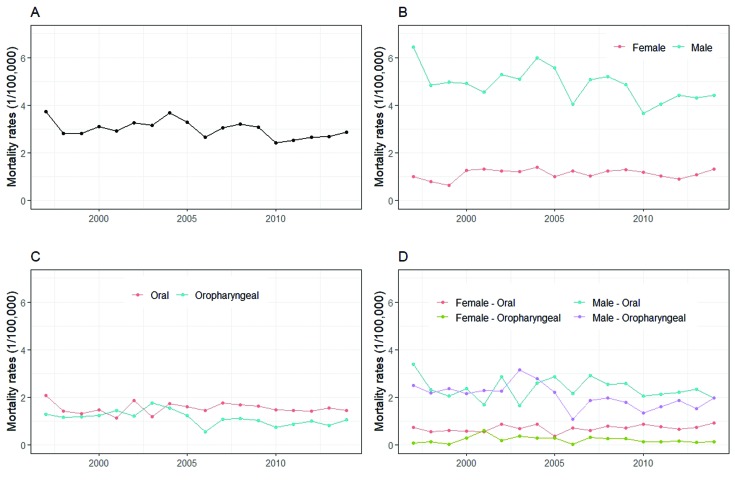
Mortality trends by oral cancer and oropharyngeal cancer in Uruguay. (A) Global mortality trend by oral cancer and oropharyngeal in Uruguay was stable between 1997 and 2014. (B) Male and female trends of mortality by oral cancer and oropharyngeal in Uruguay. (C) The analysis of mortality from oral cancer and oropharyngeal, separately remained stable over the studied period irrespective of sex. (D) The mortality rate from oral cancer and oropharyngeal cancer in men and women.

The analysis of global mortality trends by oral and oropharyngeal cancers together in Uruguay indicated stability (Fig. 1). However, the *p-value* was located in the limit (0.053); if a level of significance of 5.5% was considered, the global mortality would be decreasing. Regarding male and female mortality rates, although women's mortality rate increased from 0.51 per 100,000 in 1997 to 0.65 per 100,000 in 2014, the analysis indicates stability over the studied period (*p*=0.431). For men, rates per 100,000 went from 3.22 in 1997 to 2.20 in 2014, and this decrease was statistically significant (*p*=0.014) over the studied period (Fig. 1).

The analysis of mortality from oral cancer remained sTable over the studied period irrespective of sex (Fig. 1). As well as, mortality from oropharyngeal (*p*=0.057) (Fig. 1). The mortality rate from oral cancer was sTable over the studied period in men (*p*=0.386), while in women an increasing trend was observed (*p*=0.028) (Fig. 1). Considering oropharyngeal cancer, a decreasing trend was observed in men (*p*=0.048), while in women was observed a sTable trend (*p*=0.544). A decrease in mortality was observed in cancers situated at the base of tongue (*p*<0.001) and in the gums (*p*=0.004), remaining sTable at the other sites.

The mortality trends by oral and oropharyngeal cancer, according to province and sex demonstrated that in women, the trend remained sTable in most of the different provinces of the country, except in Lavalleja and Maldonado where there was an increase, and the mean annual change in the mortality rate was 0.1197 (*p*=0.029) and 0.0961 (*p*= 0.002) respectively. In men, a decrease was observed in the Artigas and Rivera provinces, and the mean annual change in the mortality rate was -0.5886 (*p*=0.021) and -0.2777 (*p*=0.002) respectively.

According to age group and sex, the mortality by oral and oropharyngeal cancers in women increased in two age groups: between 20 and 29 years and between 60 and 69 years (*p*=0.04 and 0.02 respectively). In men, there was a decrease in two age groups: between 40 and 49 years (*p*=0.00) and between 50 and 59 years (*p*=0.03).

The mortality rates for oral cancer, according to sex, age and geographical distribution (capital or other country provinces), are described in Table 1 and Table 2. In the capital, mortality from oral cancer in women was sTable during the studied period. However, in men aged between 40 and 49 years, there was a trend of decreasing mortality from oral cancer during the period (Table 1). In all other regions of the country, mortality from oral cancer decreased in women aged between 40 and 49 years, increased in women aged between 50 and 59 years and remained sTable in the other age groups over the studied period. In addition, there was a trend of decrease in mortality by oral cancer in men aged between 40 and 49 during the studied period (Table 2).

The mortality rates for oropharyngeal cancer, according to sex, age and geographical distribution (capital or other country provinces), are described in Table 1 and Table 2. In the capital, mortality by oropharyngeal cancer decreased in women aged between 70 and 79 years and remained sTable in the other age groups during the studied period. Also, a decrease in mortality from oropharyngeal cancer was observed in men aged 60 to 69 years living in Montevideo during the studied period (Table 1). In all other regions of the country, there was stability in the mortality rate from oropharyngeal cancer, irrespective of sex, except for the group of men aged between 70 and 79 years, where a decrease in the mortality rates during the period was observed (Table 2).

Other sociodemographic variables were analyzed such as years of education, unemployment, smoking and Gini index and no association with oral and oropharyngeal cancer mortality was observed.

**Table 1:**
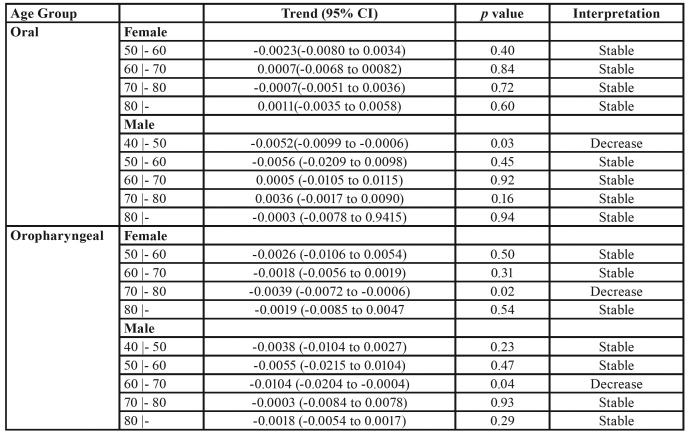
Mortality trend of oral cavity and oropharyngeal cancer in Montevideo.

**Table 2:**
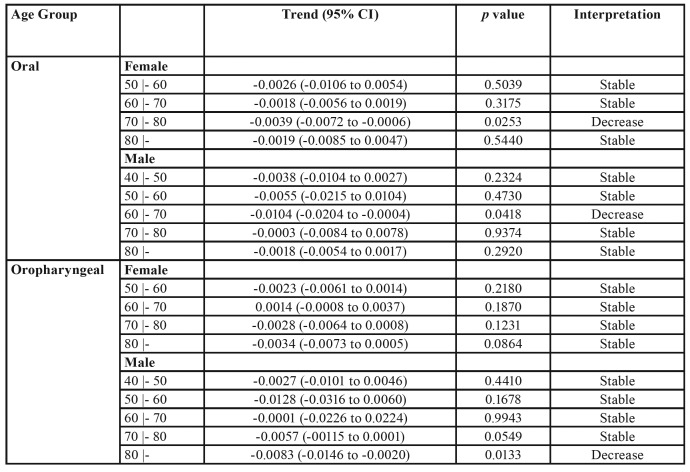
Mortality trend of oral cavity and oropharyngeal cancer in all other provinces of the country.

## Discussion

The knowledge about cancer mortality trends in a country allows medical professionals to better plan and implement new strategies for its prevention and control. In Uruguay, prior studies analyzing the trends of mortality by oral and oropharyngeal cancer were identified. They were restricted to short periods and analyzed trends of mortality from oral and oropharyngeal cancer combined. The last atlas published with data about deaths from oral and oropharyngeal cancer between 2004 and 2008 revealed mortality rates per 100,000 inhabitants, adjusted for age, of 6.46 for men and 1.16 for women (18). Analyses of longer periods provide more accurate trends including ways to monitor the effectiveness of established policies for the control of the disease and to mark the priorities. There are no such policies in Uruguay. Here, we analyzed data from 17 years of mortality, including the entire population of the country, and the results have identified interesting aspects about mortality trends in this country.

The main results showed that the global mortality by oral and oropharyngeal cancer in Uruguay has remained sTable between 1997 and 2014. Stratified analysis revealed decreasing trends of disease in men and increasing trends for women in some age groups. The international literature is somewhat controversial, but our findings are in agreement with previous studies from Mexico (19), Chile (20) and Germany (4). In Australia (5) and Canada (6) oral and oropharyngeal cancers mortality has decreased in both sexes. In Canada between 1970 and 2007, there was a significant decrease in mortality from oral cancer in both men and women. In the US and France, the mortality from oral cancer is decreasing in men (21). In Ecuador (22), China, Hungary and other countries of Central and Western Europe (21), it is increasing in both sexes.

The mortality trends from oropharyngeal cancer in Uruguay are similar to those reported in Chile (20). In Brazil, differences were detected regarding the period of time analyzed. An increase in mortality was described in a study that reported mortality between 1979 and 2002 (11). However, a decrease in all sites of the pharynx was observed for the period between 2002-2013 (12). The improvement in oral and oropharyngeal cancers mortality observed in some countries is most likely related to a decrease in tobacco use and alcohol consumption. In addition, public efforts in cancer prevention and early detection strategies along with education programs for dentists can be related to the decrease in the mortality related to these cancers (4). In Uruguay, according to the Global Survey of Adult Smoking, GAPS 2009, 25% of people 15 years old or older currently smoke (30.7% of men and 19.8% of women) with an average of 15 cigarettes per day. The measures implemented by the National Tobacco Control Programme, including the prohibition of smoking in closed places and an increase in taxes on all types of tobacco, appear to be effective in controlling the habit (15,23). In the Continuous Survey of Households of 2014, carried out by the National Institute of Statistics of Uruguay, a decreasing trend in tobacco consumption between 2011 and 2014 was observed, and the population with a high socio-educational level showed lower consumption compared to the lowest quintile (24). The Ministry of Public Health recommends the use of clinical practice guidelines to prevent breast, cervical and colorectal cancer to professionals in the country, but there are no specific strategies for the prevention of oral and oropharyngeal cancers.

Analyzing the sites separately, a decreasing trend was detected in the base of tongue and gums. Tumors of the base of the tongue, as well as others from the oropharyngeal region, may be associated with HPV infection and they have a better response to treatments and higher survival. The risk of death from HPV-related cancers in Brazil, including those of the base of the tongue, is declining, especially in men (25). Gum is a site usually more visible to clinicians with early detection of lesions. On the other hand, our study showed a certain increasing trend in the mortality of the tumors of the floor of mouth (*p-value* 0.066) and the lowest survival rate.

Our results indicated that the mortality rate from oral and oropharyngeal cancer in women increased in two age groups: between 20 and 29 years old and between 60 and 69 years of age. This trend could be due to an increase in smoking by women in recent decades. In men, there was a decrease in the age group of 30 to 59 years. Although in the present study there was no association of mortality from oral and oropharyngeal cancer with tobacco, there is strong evidence of this association in the literature (26). In Uruguay, in 2004, a law was implemented to control tobacco, and after 10 years of its application, reduced the prevalence of smoking from 32% to 20% in the general population and to 8% in young people. Although men smoke more than women, there is less abandonment of the addiction in women (23,24). While these data are encouraging, the time passed is not enough to assess the overall impact of this decrease in activity in regards to in cancer mortality since the effects of tobacco are cumulative and its impact will not appear for several years.

Regarding the geographical distribution around the country, almost no variation was observed; the principal result was a decrease in deaths in men in the Artigas and Rivera provinces. These areas are on the border with Brazil, a country with more developed oral cancer prevention policies, and in some cases, many of the Uruguayan population receives dental care or oral health education in that country which may have influenced the decrease in mortality rates (27).

Some sociodemographic variables were also analyzed, and they had no association with oral and oropharyngeal cancer mortality trends. This result does not indicate that the association does not exist, it was obtained by analyzing a model that did not include all the covariates at the same time due to multicollinearity. The variables studied were: years of education, unemployment, smoking and the Gini index, as an indicator of income inequality. The literature appointed some association between deaths from oral cancer and socioeconomic factors. In Mexico, a lower level of education and unemployment were the factors most related to deaths from oral and oropharyngeal cancer (19). Some authors deduced that the relationship between mortality from oral and oropharyngeal cancer and low socioeconomic level could be explained because that population is more exposed to some environmental variables such as tobacco use and alcohol consumption and a diet poor in fruits and vegeTables that are directly related to the development of this type of cancer. Conway *et al*. shows strong evidence that social and economic disparities indicate an independent factor in relation to mortality from this disease (28). In Uruguay, the estimation of poverty using the income method shows that there was a reduction in 2017 compared to previous years, also the Gini index in 2017 was estimated for the whole country as 0.38. A report from April 2018 by the Statics National Institute indicates that a reduction of poverty in homes across the country from 22% in 2002 to 5.5% in 2017. These data indicate that there is little inequality of income in the population of the country, thus it can be supposed that there was no association with the socioeconomic variables analyzed.

This is the first study of oral and oropharyngeal cancer mortality trends in Uruguay that analyzed both sites over a period of 17 years. One limitation of the present study was its design, because of the use of secondary data that can induce errors by subregistration or overregistration. However, the registration of mortality by cancer in Uruguay is mandatory and the data source was considered by the IARC-WHO as medium quality complete vital registration data which assures the value of our findings. Understanding the evolution of this disease allows health professionals to monitor the result of preventive actions and therapeutic strategies. The analysis of trends provides a surveillance mechanism for follow-up, but is important to consider the quality and reliability of the records.

## Conclusions

The general mortality for oral and oropharyngeal cancer in Uruguay has remained sTable for the period 1997-2014, although there is a tendency towards decreasing deaths. Important modifications were detected such as decreasing trends in men and increasing rate in women oral cavity cancer as well as a decreasing trend in the number of cancers at the base of the tongue.
